# A Smart Application for Smartphone: A Proposal to Reduce Noise Pollution for People Having Regular Tasks

**DOI:** 10.3389/fpubh.2014.00122

**Published:** 2014-08-18

**Authors:** Khaled Moustafa

**Affiliations:** ^1^Institut National de la Santé et de la Recherche Médicale, Créteil, France

**Keywords:** smartphone app, noise, regular tasks, professional meetings and noise, phone noise pollution, mobile phone disturbance

A smartphone is an important all-in-one device in today’s lifestyle, decreasing the number of other technological cumbersome and heavy-weighted tools that professionals need to carry at a time (phone, camera, GPS, laptop, book, credit card, video player, and so on). Through the installation of additional applications (apps), the list of potential uses of smartphones grows longer every day, both for professional and personal life activities. With thousands of purposeful and entertainment apps, there is something for everyone in most business and work places. Most fields in science, medicine, and society have developed apps and/or rethought their display options to ensure high quality and compatibility with smartphones screens and functionalities. The number of professional applications of smartphones is constantly increasing in all domains (medicine, engineering, linguistic, sports, etc). For example, smartphones applications have been suggested to ensure regular medication intake for chronically ill patients (1), to detect clinically active trachoma (2), to enhance the learning and behavior goals of traditional cardiac rehabilitation (3), to interpret the results of blood gas analysis, (4), to screen for HIV-related neurocognitive impairment (5), to assist clinical oncology specialists (6), and to manage and prevent exercise-induced glycemic imbalances in type 1 and type 2 diabetic patients (7, 8).

However, despite the innumerable potential uses and versatility of smartphones applications, the risk produced by, or associated with, smartphones is also increasing and alarming. In addition to addiction (9, 10) and psychology risks (11, 12), smartphones are sources of noise pollution and sound nuisance almost everywhere. Most of us have been disturbed by intempestive phone’s ringing at least tens of times in different places at social or professional occasions. The alarming messages banning the use of phones seem to be inefficient, since people tend to ignore the recommendation against the use of phones, or simply they forget to cutoff the ringtones of their phones while participating or attending social or professional meetings. In universities, for example, students and professors frequently forget that their phones are switched on during classroom sessions. Staff members, workers, and administrators, who have regular tasks, in any domain, also frequently forget that their phones are ringing and disturbing audience. To reduce such recurrent noise disturbances, I would suggest a proposal for developing a smartphone application that would switch the ringtone On/Off automatically at regular intervals to reduce the noise pollution associated with smartphones, particularly for people having regular tasks over the time. The suggestion would involve that each institution, establishment, or corporation having regular, repetitive tasks, such as systematic professional meetings, activities, or education sessions, such as universities, hospitals, banks, churches, mosques, national libraries, etc., can develop a smartphone application whose function will be to automatically mute or silent the phones during the regular activity, at, for example, customizable intervals (for ex. 10, 15, 30 min) before and after the regular meetings or activities. At the end of the set up interval, the application should automatically turn the ringtone into the normal mode (ringing). Doing so, the risk of phone disturbances would be reduced considerably.

People or institutions having regular tasks or activities whose members could be disturbed intempestively by phone ringings can develop an application to automatically silent the smartphone a few minutes before to a few minutes after, spanning the regular task period (for instance, the time of meeting, seminar, lecture, …etc). Universities and colleges, for examples, can develop such an application for their students and professors by disciplines, classes, or schedules to prevent student’s phones from ringing during the classroom lectures. The mute options could be set up, for example, to 5, 10, 15, or 30 min (less or more) before and after the onset of classroom sessions. Timetables’ details could be further explored for each matter, class, seasons, holidays…etc, to make the application as smart as possible to function only during the desired collective time to avoid any undesirable troublesome noise. National libraries can also develop such an application and make it available for their readers to avoid any noise during the opening hours. Believers (Jews, Christians, Muslims, Buddhists, …, etc.) who have regular prayers at regular timings would also appreciate an automatic cutoff of their smartphones at the prayer times, 10, 15, or 20 min before and after each prayer.

Since omission or forgetfulness is a human attribute, an automatic deactivation/reactivation of the ringing mode at customizable, regular intervals would certainly be highly appreciable by all professionals and people looking to concentrate and decent environment to perform their works. The sole caveat, in my view, with such an application would be that some users may not activate or download the application. However, most people would appreciate the automatically reduced disturbance by such an option, which should encourage them to use it and activate it. People who are afraid that they might forget that their phones are switched on during their intellectual activities would strongly approve the proposed idea. The implementation of such an application for an automatic mute/sound mode in a smartphone would add an extra “smartness” to the smartphones for a great number of users and would reduce a public health issue related to auditory disturbances. The development and use of such an application should be free of charge to reduce noise pollution for mutual interests of phone users and audience.

## Conflict of Interest Statement

The author declares that the research was conducted in the absence of any commercial or financial relationships that could be construed as a potential conflict of interest.
